# Variables predicting weaning outcome in prolonged mechanically ventilated tracheotomized patients: a retrospective study

**DOI:** 10.1186/s40560-020-00437-4

**Published:** 2020-02-21

**Authors:** Alessandro Ghiani, Joanna Paderewska, Alexandros Sainis, Alexander Crispin, Swenja Walcher, Claus Neurohr

**Affiliations:** 1 Department of Pneumology and Respiratory Medicine, Schillerhoehe Lung Clinic (Robert Bosch Hospital GmbH), Solitudestr. 18, 70839 Gerlingen, Germany; 2 Athens, Greece; 3 IBE – Institute for Medical Information Processing, Biometry and Epidemiology, Ludwig-Maximilians-University (LMU), Marchioninistr. 15, 81377 Munich, Germany

**Keywords:** Respiration, Mechanical ventilation, Tracheostomies, Ventilator weaning, Work of breathing, Mechanical power

## Abstract

**Background:**

Several studies have assessed predictors of weaning and extubation outcome in short-term mechanically ventilated patients, but there are only few studies on predictors of weaning from prolonged mechanical ventilation.

**Methods:**

Retrospective, single-center, observational study at a specialized national weaning center in Germany. Patients’ medical records were reviewed to obtain data on demographics, comorbidities, respiratory indices, and the result of a prospectively documented, standardized spontaneous breathing trial (SBT) upon admission to the weaning center. Respiratory indices assessed were the ventilatory ratio (VR) and parameters derived from calculated mechanical power (MP). Predictors associated with failure of prolonged weaning and failure of the SBT were assessed using a binary logistic regression model.

**Results:**

A total of 263 prolonged mechanically ventilated, tracheotomized patients, treated over a 5-year period were analyzed. After 3 weeks of mechanical ventilation, patients with unsuccessful weaning failed a SBT more frequently and showed significantly increased values for inspiratory positive airway pressure, driving pressure, VR, absolute MP, and MP normalized to predicted body weight and dynamic lung-thorax compliance (LTC-MP). In the logistic regression analyses, variables independently correlated with weaning failure were female gender (adjusted odds ratio 0.532 [95% CI 0.291–0.973]; *p* = 0.040), obesity (body mass index ≥ 30 kg/m^2^) (2.595 [1.210–5.562]; *p* = 0.014), COPD (3.209 [1.563–6.589]; *p* = 0.002), LTC-MP (3.470 [1.067–11.284]; *p* = 0.039), P_a_CO_2_ on mechanical ventilation (1.101 [95% CI 1.034–1.173]; *p* = 0.003), and failure of the SBT (4.702 [2.250–9.825]; *p* < 0.001). In addition, female gender (0.401 [0.216–0.745]; *p* = 0.004), LTC-MP (3.017 [1.027–8.862]; *p* = 0.046), and P_a_CO_2_ on mechanical ventilation (1.157 [1.083–1.235]; *p* < 0.001) were independent risk factors for an unsuccessful SBT.

**Conclusions:**

In the present study, the derived predictors of weaning point to a crucial role of the workload imposed on respiratory muscles during spontaneous breathing. Mechanical power normalized to lung-thorax compliance was independently correlated with weaning outcome and may identify patients at high risk for weaning failure.

## Background

Intubation with mechanical ventilation is a life-saving procedure for patients presenting with acute respiratory failure. However, since unnecessarily prolonged mechanical ventilation is associated with increased mortality [1, 2], weaning from the ventilator, which accounts for approximately 40–50% of total ventilation time [3, 4], should begin as early as possible. In order to provide structure and to safely accelerate the weaning process, a classification into six stages has been proposed [5], with clinical parameters and tests (including weaning trials) at each of the stages that may help predict successful transition to the next stage (and eventually to extubation) [6]. If a patient experiences prolonged weaning [5], undergoes multiple unsuccessful weaning trials, or has to be reintubated despite using non-invasive ventilation to prevent post-extubation respiratory failure, tracheostomy is indicated, often followed by transferal to specialized weaning and home ventilation centers [7–10]. At such centers, since the pathophysiological basis of prolonged weaning is an imbalance between respiratory muscle load and capacity [11, 12], the aim is to both reconditioning the respiratory pump, while at the same time reducing workload imposed during spontaneous breathing. So far, few studies have examined predictors of prolonged weaning in such patients [13–18].

The aim of the present study is to derive such predictors from demographics, clinical characteristics, comorbidities, respiratory indices, and from the results of a standardized first spontaneous breathing trial (SBT) upon admission to the weaning center.

## Methods

This was a retrospective, single-center, observational cohort study conducted at a national weaning center in Germany. The 12-bed ward was established in 2006 and is part of the specialized lung clinic at Schillerhoehe (Gerlingen). The weaning unit is equipped to provide invasive (by tracheostomy tube) and non-invasive ventilatory support. Main preconditions for referral to the unit are hemodynamic stability without need for vasopressors or inotropic agents, wakefulness without need for permanent sedation, and invasive mechanical ventilation by tracheostomy tube with a positive end-expiratory pressure (PEEP) less than 10 cm H_2_O and a fraction of inspired oxygen (F_i_O_2_) less than 0.6.

The study was approved by the local ethics committee, and the need for informed consent was waived.

### Patient selection

**Fig. 1:**
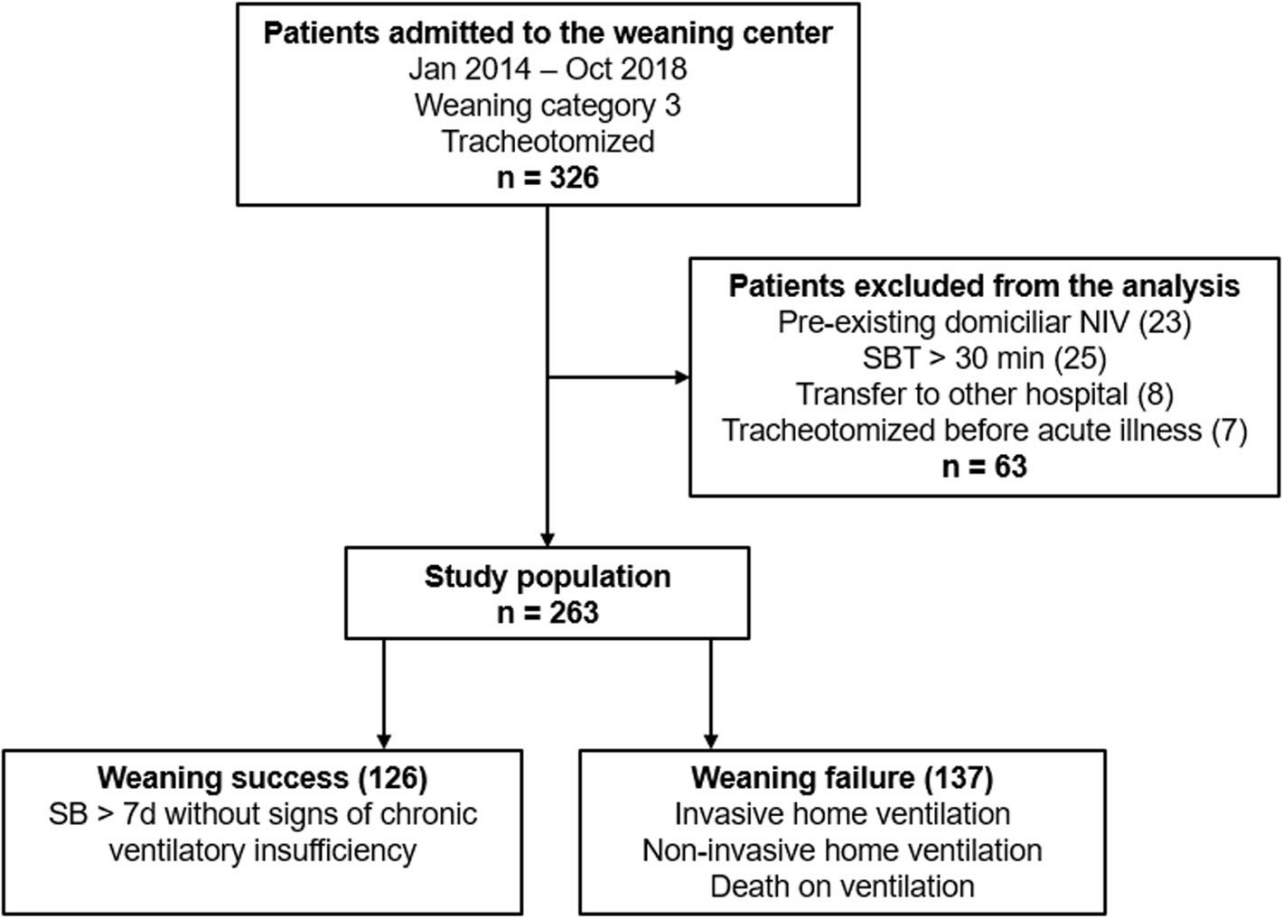
Patient selection. Weaning category 3 refers to the statement from Boles and colleagues [5]. **N**IV, non-invasive ventilation; SBT, spontaneous breathing trial; SB, spontaneous breathing

All consecutive patients transferred to the weaning center between January 2014 and October 2018 were identified. Patients were included in the analyses if they were referred because of evident failure to wean from mechanical ventilation, and if they met the criteria of prolonged weaning, classified as category 3 as defined by Boles and colleagues (i.e., failed at least three weaning attempts or required more than 7 days of weaning after the first SBT). Patients classified as category 1 (simple weaning: patients who proceeded from initiation of weaning to successful extubation on the first attempt without difficulty) or category 2 (difficult weaning: patients who fail initial weaning and require up to three SBT or as long as 7 days from the first SBT to achieve successful weaning) were excluded from the analyses [5]. We analyzed data only from patients whose first SBT upon admission to the weaning center was performed over a period of 30 min. Furthermore, patients were excluded from our study if a tracheostomy or non-invasive home ventilation (NIV) existed before the acute illness, or if the patient was subsequently transferred to another hospital prior to completion of the weaning process (Fig. 1).

### Ventilator weaning

Weaning was systematically performed according to the recommendations of Boles and colleagues [5] and the national guidelines on prolonged weaning [19]. The process included protocol-based increasing periods of unassisted breathing through a tracheostomy collar (weaning trials), usually starting with a 30 min SBT, with the duration increasing by approximately 2 h per day. In the intervals between SBT, all patients were mechanically ventilated in the assisted-controlled mode (Vivo 50, Breas Medical AB, Moelnlycke, Sweden) in order to recover from the imposed work of breathing during SBT [20]. The overall weaning program included nutritional support, proactive physiotherapy, and optimal therapy of comorbidities, with use of sedatives avoided.

Patients were classified at the end of the weaning process into two categories: patients with successful weaning (weaning success) and those with unsuccessful weaning (weaning failure). Successful weaning was defined as spontaneous breathing for more than 7 days without concomitant clinical or laboratory signs of chronic ventilatory insufficiency. Unsuccessful weaning was either death in the course of weaning or the transition to permanent non-invasive (by face mask) or invasive (by tracheostomy tube) home ventilation due to chronic ventilatory insufficiency. Chronic ventilatory insufficiency was defined as recurrent hypercapnia during the daily weaning trials, preventing the extension of spontaneous respiration, or hypercapnia occurring within 7 days after weaning completion, requiring resumption of mechanical ventilation.

### Spontaneous breathing trials

All weaning trials were standardized and were documented prospectively. The first SBT upon admission to the weaning center occurred as soon as the criteria for weaning readiness were met, which were adequate oxygenation with a PEEP ≤ 8 cmH_2_O, hemodynamic stability without the need for vasopressors or inotropic agents, and normocapnia (P_a_CO_2_ ≤ 45.0 mmHg) on mechanical ventilation. At the time of the SBT, patients were not sedated.

For these weaning trials, patients were placed in the semi-recumbent position, ventilator variables were recorded, and an arterial blood gas analysis (aBGA) was performed. The patient was then disconnected from the ventilator and breathed room air for 30 min through a T-piece with oxygen admixture to ensure sufficient oxygenation (S_p_O_2_ ≥ 92%). The first SBT was performed under the supervision of a respiratory therapist, and vital signs were continuously monitored to immediately detect respiratory distress. Another aBGA was performed at the end of the SBT and, if possible, also in the event of premature termination of SBT due to respiratory distress.

Failure of SBT was defined as the occurrence of clinical signs of respiratory failure (agitation and anxiety, diaphoresis, breathing frequency > 30/min, tachycardia >130 bpm, systolic blood pressure > 160 mmHg, or S_p_O_2_ < 88%) or changes in blood gas values consistent with ventilatory failure (hypercapnia [P_a_CO_2_ > 45.0 mmHg] with or without respiratory acidosis [pH < 7.35]) [5].

### Data collection

Data were retrospectively collected from referral letters and the hospital’s electronic medical record and chart system. Patients’ baseline characteristics on admission, such as demographic data and comorbidities were collected.

Ventilator variables included inspiratory positive airway pressure (IPAP), PEEP, oxygen flow on mechanical ventilation, breathing frequency (BF), inspiratory tidal volume (VTi), and minute ventilation (VE). The following were then calculated from the collected parameters: driving pressure (DP), dynamic lung-thorax compliance (LTC) [21], mechanical power (MP) [22–24], and ventilatory ratio (VR) [25, 26]. MP indices were calculated by normalizing absolute values to predicted body weight (PBW-MP), to LTC (LTC-MP) [27], and to minute ventilation (VE-MP).

Further details on definitions of ventilator variables, respiratory indices, and weaning outcome measures can be found in the online supplement [see Additional file 1].

### Statistical analyses

All analyses were exploratory, without formal sample size planning. Data are reported as mean and standard deviation for continuous variables and number and percentages for categorical variables.

Descriptive and frequency statistics were used to summarize demographics and baseline characteristics. Differences between groups in categorical variables were analyzed using the Chi-squared test or Fisher’s exact test, as appropriate. Student’s *t* test was used to examine differences in continuous variables.

Predictors for unsuccessful weaning or unsuccessful SBT, which were derived from patients’ clinical characteristics, comorbidities, respiratory indices, and the result of first SBT, were modeled using a binary logistic regression analysis. Age and gender were forced into the model, with the remaining predictors introduced to the model using a *P* value-based forward selection at the alpha level of five percent.

We considered *P* < 0.05 to be statistically significant for all tests performed.

## Results

Between January 2014 and October 2018, 326 patients were screened for eligibility, and a total of 263 patients (80.7%) were included in the analyses. Individuals with weaning failure were more frequently female, had more ventilator days on admission, and there was a larger proportion of patients with obesity (body mass index ≥ 30 kg/m^2^), chronic obstructive pulmonary disease (COPD) as a comorbidity, and acute exacerbation of COPD as the cause of respiratory failure. The remaining parameters were not significantly different between the two groups (Table 1).

**Table 1:** Clinical characteristics on admission to the weaning center

	All patients (*n* = 263)	Weaning success (*n* = 126)	Weaning failure (*n* = 137)	*P* value^*a*^
Clinical characteristics				
Age (years)	70.9 (± 11.0)	71.0 (± 11.4)	70.7 (± 10.6)	0.404^*b*^
Gender (male)	161 (61.2)	87 (69.0)	74 (54.0)	**0.012**^***c***^
Body mass index (kg/m^2^)	26.4 (± 6.2)	25.1 (± 4.4)	27.7 (± 7.3)	**< 0.001**^***b***^
Obesity (BMI ≥ 30 kg/m^2^)	54 (20.5)	16 (12.7)	38 (27.7)	**0.003**^***c***^
Smoking history	120 (45.6)	50 (39.7)	70 (51.1)	0.063^*c*^
APACHE-II (points)	17.7 ( 5.1)	17.6 (± 4.6)	17.9 (± 5.5)	0.350^*b*^
Albumin (g/dL)	2.0 (± 0.5)	2.0 (± 0.5)	2.0 (± 0.5)	0.190^*b*^
Ventilator days on admission (days)	26.7 (± 21.6)	24.2 (± 17.2)	29.0 (± 24.8)	**0.034**^***b***^
Time from intubation to tracheostomy (days)	10.6 (± 6.7)	10.7 (± 6.5)	10.4 (± 6.9)	0.395^*b*^
ECLA	11 (4.2)	8 (6.3)	3 (2.2)	0.092^*c*^
Cause of acute respiratory failure				
Pneumonia	77 (29.3)	42 (33.3)	35 (25.5)	0.166^*c*^
Surgery	74 (28.1)	38 (30.2)	36 (26.3)	0.484^*c*^
Sepsis (including septic shock)	29 (11.0)	14 (11.1)	15 (10.9)	0.967^*c*^
Acute exacerbation of COPD	26 (9.9)	2 (1.6)	24 (17.5)	**< 0.001**^***c***^
Cardiopulmonary resuscitation	22 (8.4)	11 (8.7)	11 (8.0)	0.837^*c*^
Cardiac failure	8 (3.0)	4 (3.2)	4 (2.9)	0.904^*c*^
Trauma	5 (1.9)	3 (2.4)	2 (1.7)	0.673^*d*^
Other	22 (8.4)	12 (9.5)	10 (7.3)	0.515^*c*^
Comorbidities				
Charlson comorbidity index (points)	6.6 (± 2.4)	6.5 (± 2.4)	6.7 (± 2.5)	0.268^*b*^
Coronary artery disease	89 (33.8)	43 (34.1)	46 (33.6)	0.925^*c*^
COPD	65 (24.7)	15 (11.9)	50 (36.5)	**< 0.001**^***c***^
Diabetes mellitus	82 (31.2)	39 (31.0)	43 (31.4)	0.939^*c*^
Chronic heart failure	53 (20.2)	22 (17.5)	31 (22.6)	0.297^*c*^
Renal insufficiency (GFR < 60 mL/min)	74 (28.1)	30 (23.8)	44 (32.1)	0.134^*c*^
Hemodialysis on admission	34 (12.9)	12 (9.5)	22 (16.1)	0.115^c^
Malignancy	37 (14.1)	22 (17.5)	15 (10.9)	0.129^*c*^
Hepatopathy	13 (4.9)	8 (6.3)	5 (3.6)	0.313^*c*^
Interstitial lung disease	12 (4.6)	7 (5.6)	5 (3.6)	0.459^*c*^
Neuromuscular disease	12 (4.6)	7 (5.6)	5 (3.6)	0.459^*c*^

Continuous variables are presented as mean values (± standard deviation); categorical variables are presented as number (%)

^*a*^*P* value for differences between patients with weaning success and weaning failure

^*b*^Student’s *t* test

^*c*^Chi-squared test

^*d*^Fisher’s exact test

*BMI* body mass index, *APACHE-II* Acute Physiology and Chronic Health Evaluation II score, *ECLA* extracorporeal lung assistance (in acute respiratory failure), *COPD* chronic obstructive pulmonary disease, *GFR* glomerular filtration rate

### Spontaneous breathing trials

All patients reached normocapnia (P_a_CO_2_ ≤ 45 mmHg) on mechanical ventilation; 221 patients (84%) were on controlled ventilation prior to the SBT, with no significant differences between the two groups (82% and 86% in the weaning success and failure groups, respectively; *p* = 0.757). Patients with weaning failure had significantly higher values for IPAP, PEEP, DP, and VR than those with successful weaning. Similarly, calculated absolute MP, derived PBW-MP, LTC-MP and VE-MP, and P_a_CO_2_ on mechanical ventilation were all significantly higher in these patients, who also failed their first SBT more frequently (Table 2).

**Table 2:** Ventilator variables, respiratory indices and results of first spontaneous breathing trial

Ventilator variables, respiratory indices, and results of first SBT	All patients (*n* = 263)	Weaning success (*n* = 126)	Weaning failure (*n* = 137)	*P* value^*a*^
Ventilator variables				
IPAP (cmH_2_O)	23.4 (± 4.8)	21.7 (± 4.4)	24.9 (± 4.6)	**< 0.001**^***b***^
PEEP (cmH_2_O)	5.9 (± 1.2)	5.6 (± 0.8)	6.1 (± 1.5)	**< 0.001**^***b***^
DP (cmH_2_O)	17.5 (± 4.5)	16.1 (± 4.2)	18.7 (± 4.5)	**< 0.001**^***b***^
VTi (mL)	534 (± 82)	535 (± 81)	533 (± 82)	0.416^*b*^
BF (breaths/min)	16.2 (± 2.5)	16.1 (± 2.6)	16.3 (± 2.4)	0.288^*b*^
VE (L/min)	8.6 (± 1.8)	8.6 (± 1.7)	8.7 (± 1.9)	0.306^*b*^
Oxygen flow on MV (L/min)	2.6 (± 1.8)	2.5 (± 1.7)	2.7 (± 1.8)	0.123^*b*^
Respiratory indices				
LTC (mL/cmH_2_O)	32.8 (± 10.8)	35.5 (± 11.1)	30.3 (± 9.9)	**< 0.001**^***b***^
Mechanical power (J/min)	19.8 (± 5.9)	18.4 (± 5.3)	21.2 (± 6.1)	**< 0.001**^***b***^
PBW-MP (J/min/kg PBW × 10^-3^)	277 (± 87)	252 (± 79)	299 (± 88)	**< 0.001**^***b***^
LTC-MP (J/min × cmH_2_O/mL × 10^-3^)	685 (± 348)	585 (± 297)	777 (± 366)	**< 0.001**^***b***^
VE-MP (J/L)	2.29 (± 0.47)	2.13 (± 0.43)	2.44 (± 0.45)	**< 0.001**^***b***^
Ventilatory ratio	1.14 (± 0.27)	1.07 (± 0.26)	1.20 (± 0.28)	**< 0.001**^***b***^
First SBT				
Time from admission to first SBT (days)	2.5 (± 4.2)	2.1 (± 4.2)	2.9 (± 4.3)	0.056^*b*^
Hemoglobin on first SBT (g/dL)	8.8 (± 1.2)	8.7 (± 1.2)	8.9 (± 1.2)	0.085^*b*^
P_a_CO_2_ on aBGA				
P_a_CO_2_ on MV pre-SBT (mmHg)	35.7 (± 5.0)	34.2 (± 4.8)	37.0 (± 4.8)	**< 0.001**^***b***^
P_a_CO_2_ post-SBT (mmHg)	39.1 (± 6.5)	36.8 (± 5.7)	41.5 (± 6.3)	**< 0.001**^***b***^
∆P_a_CO_2_ (pre-/post-SBT)	3.4 (5.5)	2.5 (± 4.8)	4.4 (± 5.9)	**0.005**^***c***^
pH on aBGA				
pH on MV pre-SBT	7.48 (± 0.05)	7.50 (± 0.05)	7.47 (± 0.05)	**< 0.001**^***b***^
pH post-SBT	7.45 (± 0.05)	7.47 (± 0.05)	7.43 (± 0.05)	**< 0.001**^***b***^
∆pH (pre-/post-SBT)	− 0.03 (± 0.05)	− 0.03 (± 0.05)	− 0.04 (± 0.06)	**0.025**^***b***^
P_a_O_2_ on aBGA				
P_a_O_2_ on MV pre-SBT (mmHg)	81.4 (± 18.7)	81.9 (± 17.4)	80.9 (± 19.9)	0.325^*b*^
P_a_O_2_ post-SBT (mmHg)	74.3 (± 17.2)	76.3 (± 18.9)	72.2 (± 15.0)	**0.036**^***b***^
∆P_a_O_2_ (pre-/post-SBT)	6.9 (± 22.3)	6.2 (± 24.4)	7.7 (± 19.8)	0.293^*b*^
Duration of first SBT (min)	27.0 (± 7.5)	28.8 (± 4.9)	25.2 (± 9.0)	**< 0.001**^***b***^
Reason for failure of first SBT	72 (27.4)	12 (9.5)	60 (43.8)	**< 0.001**^***c***^
Acidosis post-SBT	10 (3.8)	2 (1.6)	8 (5.8)	0.072^*d*^
Hypercapnia post-SBT	37 (14.1)	6 (4.8)	31 (22.6)	**< 0.001**^***c***^
Premature termination of SBT	42 (16.0)	8 (6.3)	34 (24.8)	**< 0.001**^***c***^

Continuous variables are presented as mean values (± standard deviation); categorical variables are presented as number (%)

^*a*^*P* value for differences between patients with weaning success and weaning failure

^*b*^Student’s *t* test

^*c*^Chi-squared test

^*d*^Fisher’s exact test

*IPAP* inspiratory positive pressure ventilation, *PEEP* positive end-expiratory pressure, *DP* driving pressure, *VTi* inspiratory tidal volume, *BF* breathing frequency, *VE* minute ventilation, *MV* mechanical ventilation, *LTC* dynamic lung-thorax compliance, *MP*, mechanical power, *J* joule, *PBW-MP* mechanical power normalized to predicted body weight, *PBW* predicted body weight, *LTC-MP* mechanical power normalized to dynamic lung-thorax compliance, *VE-MP* mechanical power normalized to minute ventilation, *aBGA* arterial blood gas analysis, *SBT* spontaneous breathing trial

### Weaning outcome measures

In the weaning failure group, the duration of weaning and length of hospital stay were significantly increased, and there were more patients with long-term oxygen therapy (LTOT) on discharge than in the successful weaning group (Table 3). Hospital mortality in all patients was 14.4% and did not differ significantly between the two groups. Death on mechanical ventilation occurred in 9.5% of patients with unsuccessful weaning after a mean of 24 days upon first SBT (median 21 days, range 3–58 days).

**Table 3:** Weaning outcome

Weaning outcome measures	All patients (*n* = 263)	Weaning success (*n* = 126)	Weaning failure (*n* = 137)	*P* value^*a*^
Weaning success	126 (47.9)	126 (100)	–	–
Weaning failure	137 (52.1)	–	137 (100.0)	–
HMV-NIV	50 (19.0)	–	50 (36.5)	–
HMV-IMV	74 (28.1)	–	74 (54.0)	–
Death on MV	13 (4.9)	–	13 (9.5)	–
Weaning duration from first SBT (days)	22.9 (± 16.1)	19.0 (± 11.7)	26.6 (± 18.5)	**< 0.001**^*b*^
Duration of mechanical ventilation (days)	52.2 (± 28.6)	45.2 (± 22.1)	58.6 (± 32.1)	**< 0.001**^*b*^
Decannulation	135 (51.3)	85 (67.5)	50 (36.5)	**< 0.001**^*c*^
SB on discharge from hospital (hours per day)^*d*^	16.1 (± 8.9)	21.9 (± 6.5)	10.9 (± 7.5)	**< 0.001**^*b*^
LTOT on discharge	183 (78.9)	74 (63.8)	109 (94.0)	**< 0.001**^*c*^
Weaning unit-LOS (days)	48.3 (± 28.5)	40.8 (± 21.7)	55.1 (± 32.0)	**< 0.001**^*b*^
Hospital-LOS (days)	52.8 (± 28.6)	45.6 (± 22.2)	59.5 (± 32.0)	**< 0.001**^*b*^
Hospital mortality	38 (14.4)	13 (10.3)	25 (18.2)	0.068^*c*^

Continuous variables are presented as mean values (± standard deviation); categorical variables are presented as number (%)

^*a*^*P* value for differences between patients with weaning success and weaning failure

^*b*^Student’s *t* test

^*c*^Chi-squared test

^*d*^Values for deceased patients were set at 0 h

*HMV-NIV* home mechanical ventilation-non-invasive mechanical ventilation, *HMV-IMV* home mechanical ventilation-invasive mechanical ventilation, *IMV* invasive mechanical ventilation, *SB* spontaneous breathing, *LTOT* long-term oxygen therapy, *SBT* spontaneous breathing trial, *LOS* length of stay

### Results of logistic regression analyses

Variables independently related to weaning failure were female gender, obesity, COPD, LTC-MP, P_a_CO_2_ on mechanical ventilation, and failure of the first SBT upon admission to the weaning center (Table 4).

**Table 4:** Variables associated with failure of prolonged weaning and failure of spontaneous breathing trial—results of binary logistic regression model

Variables	Failure of prolonged weaning		Failure of first SBT	
	OR (95% CI)	*P* value	OR (95% CI)	*P* value
Gender (male)	0.532 (0.291–0.973)	**0.040**	0.401 (0.216–0.745)	**0.004**
Obesity (BMI > 30 kg/m^2^)	2.595 (1.210–5.562)	**0.014**	–	n.s.
COPD	3.209 (1.563–6.589)	**0.002**	–	n.s.
LTC-MP	3.470 (1.067–11.284)	**0.039**	3.017 (1.027–8.862)	**0.046**
P_a_CO_2_ on MV	1.101 (1.034–1.173)	**0.003**	1.157 (1.083–1.235)	**< 0.001**
Failed first SBT	4.702 (2.250–9.825)	**< 0.001**	–	n.a.

*SBT* spontaneous breathing trial, *OR* odds ratio, *95% CI* 95% confidence interval, *n.s.* not significant, *BMI* body mass index, *COPD* chronic obstructive pulmonary disease, *LTC-MP* mechanical power normalized to dynamic lung-thorax compliance, *n.s.* not significant, *MV* mechanical ventilation, *n.a.* not applicable

**Fig. 2:**
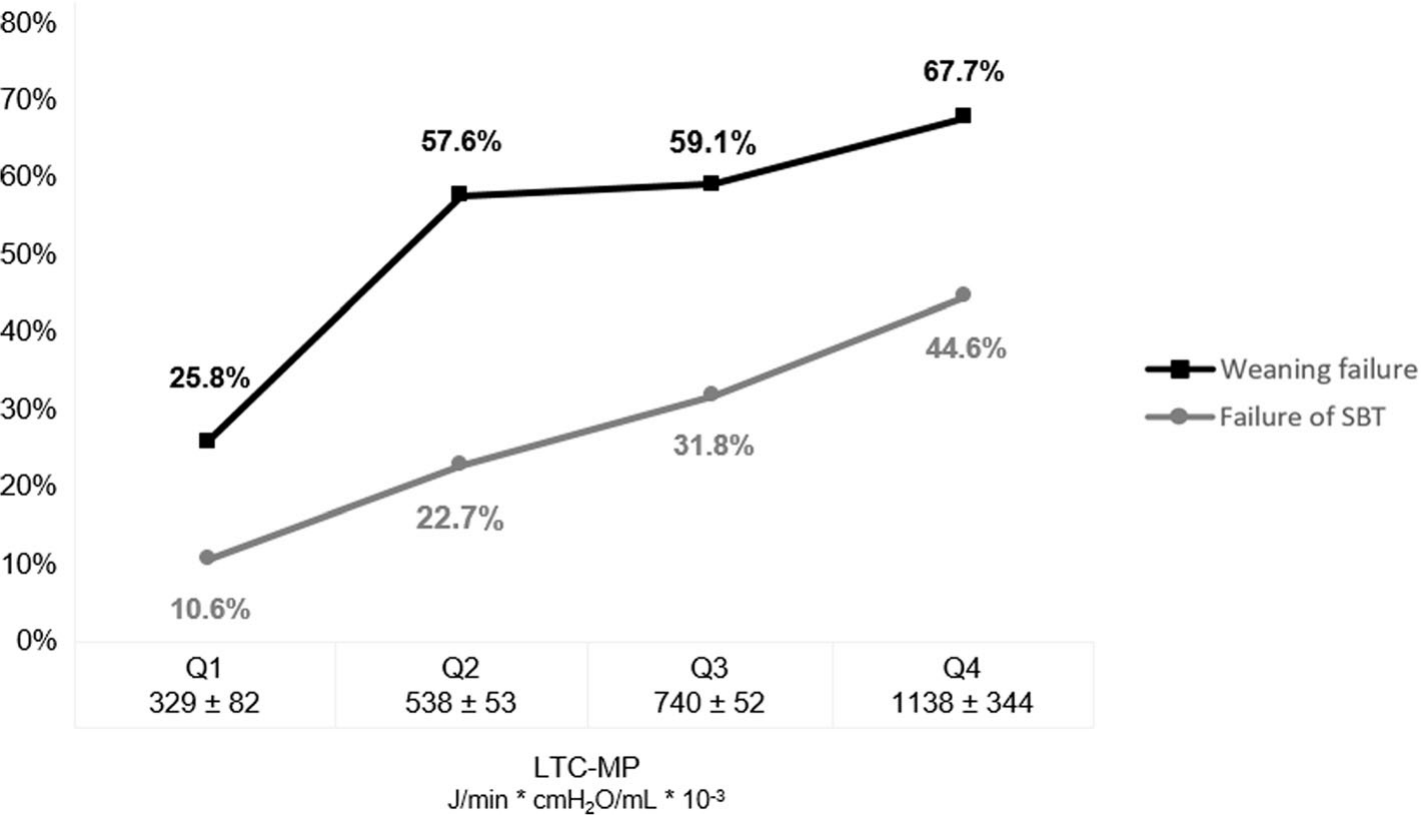
Failure rates of prolonged weaning and SBT in patients classified by quartiles of LTC-MP**.** Failure rates are presented as percentage (%), LTC-MP as mean values, and standard deviation. SBT, spontaneous breathing trial; LTC-MP, mechanical power normalized to dynamic lung-thorax compliance; Q1-4, patients classified by quartiles 1-4 of LTC-MP

In addition, female gender, LTC-MP (Fig. 2), and P_a_CO_2_ on mechanical ventilation were independent risk factors for failure of the first SBT.

Weaning failure was more common in obese than in non-obese patients (70.4% vs. 47.4%, respectively; *p* = 0.003), who also had significantly higher values for MP (22.4 ± 6.0 vs. 19.2 ± 6.7 J/min; *p* < 0.001), PBW-MP (328 ± 95 vs. 263 ± 80 J/min/kg PBW × 10^-3^; *p* < 0.001), LTC-MP (768 ± 339 vs. 664 ± 347 J/min × cmH_2_O/ml × 10^-3^; *p* = 0.025), and VR (1.23 ± 0.28 vs. 1.11 ± 0.27; *p* = 0.004). In contrast, there was no difference in P_a_CO_2_ on mechanical ventilation (35.4 ± 5.2 vs. 35.6 ± 5.2 mmHg; *p* = 0.123).

Based on the median P_a_CO_2_ before the first SBT, the total population was also divided into patients with P_a_CO_2_ < vs. ≥ 36 mmHg. Patients with lower P_a_CO_2_ were less likely to have unsuccessful weaning (40.2% vs. 62.4%; *p* < 0.001) and a failed first SBT (16.4% vs. 36.9%; *p* < 0.001) and showed significantly lower values for LTC-MP (624 ± 304 vs. 738 ± 374 J/min × cmH_2_O/ml × 10^-3^; *p* = 0.004) and for VR (1.02 ± 0.22 vs. 1.24 ± 0.28; *p* < 0.001).

Table S1 compares clinical characteristics and outcomes between female and male patients (see Additional file 1). Females failed the first SBT more frequently (35.3% vs. 22.4%; *p* = 0.023), and weaning failure occurred in a significantly higher percentage of patients (61.8% vs. 46.0%; *p* = 0.012).

## Discussion

The present study identified clinical predictors of prolonged weaning in long-term mechanically ventilated, tracheotomized patients, treated at a specialized weaning center, and the results point to a crucial role of the workload imposed on respiratory muscles of such patients. The results of the study can be summarized as follows. First, variables independently associated with failure of prolonged weaning were female gender, obesity, COPD as a comorbidity, LTC-MP, P_a_CO_2_ on mechanical ventilation, and failure of the first SBT upon admission to the weaning center. Secondly, female gender, LTC-MP, and P_a_CO_2_ on mechanical ventilation were also independently correlated with an unsuccessful first SBT.

The mechanical power of artificial ventilation describes the energy transmitted from the ventilator to the respiratory system. MP can be obtained mathematically by the “power equation,” derived from the original equation of motion, unifying all determining ventilator variables, or experimentally by analysis of the respiratory system pressure-volume curve (P-V curve) [22]. The energy transmitted largely serves to overcome elastic recoil and airway resistance of the respiratory system, although some dissipates into the tissue of the lungs and the thoracic cage, with the component dissipating into the tissue of the lungs believed to be responsible for the development of ventilator-induced lung injury (VILI) [27]. In contrast, the pathophysiological basis for prolonged weaning is thought to be reduced respiratory muscle capacity [11, 12], accompanied by increased load of the respiratory pump due to changes in the mechanical properties of the lungs (infiltrates, fibrosis, emphysema) and the thoracic cage (pleural effusion, increased abdominal pressure, and anasarca of the chest wall), that may be either reversible (by therapeutic intervention) or irreversible (e.g., due to pre-existing comorbidities). Patients’ measured work of breathing (WOB) during spontaneous respiration, which is identical to the mechanical power generated by respiratory muscles, has proved to be a predictor of weaning [28–30]; however, since WOB requires esophageal pressure measuring, the procedure is complex, impractical, and rarely used in routine practice. In contrast, energy delivered by the ventilator can be continuously calculated at the bedside and may be even directly displayed on the ventilator. Since the energy transmitted by the ventilator was found to be an independent variable related to weaning failure, corresponding to a state of chronic ventilatory insufficiency in the course of prolonged mechanical ventilation, it may at least be hypothesized, that at a particular P_a_CO_2,_ the MP transferred on mechanical ventilation would be correlated to a patients’ WOB. In the present study, patients in the weaning failure group experienced significantly elevated MP values, mainly due to higher values for IPAP, indicating that with increasing MP, in order to secure adequate gas exchange, the likelihood of long-term ventilator dependency increases. LTC-MP describes the energy required to dilate the lungs and thoracic cage, and the present results indicate that this is crucial in prolonged weaning. In fact, therapeutic interventions during weaning aim at improving respiratory system compliance and resistance, thereby reducing a patient’s WOB. In contrast, PBW-MP relates the transmitted energy to lung volume, which appears to be critical in the development of VILI in patients with acute respiratory distress syndrome [27].

Changes in respiratory mechanics in obese patients comprise reduced functional residual capacity (FRC), located in the shallow lower part of the P-V curve, and basal atelectasis due to elevated abdominal pressure, resulting in reduced LTC and increased WOB [31]. In addition, there is an approximate 1.5-fold increase in oxygen consumption even at rest with simultaneous excessive CO_2_ production. This may help to explain the observed increased VR in such patients in the present study, equivalent to increased ventilatory demands, so indirectly contributing to the observed increased MP. These results are consistent with previous work, in which obesity has been identified as independently associated with failure of prolonged mechanical ventilation in tracheotomized patients treated at specialized long-term acute care facilities (LTAC) [17]. Similarly, in intubated patients, obesity is independently associated with increased extubation failure risk, and prophylactic use of non-invasive ventilation to reduce patients’ WOB has proved to prevent reintubation [32].

Just as in patients with obesity, the presence of COPD is characterized by specific changes in respiratory mechanics, primarily bronchial obstruction accompanied by lung hyperinflation. With increased respiratory rates, leading to dynamic hyperinflation, the FRC moves to the flat upper margin of the P-V curve. This results in increased WOB due to reduced LTC, together with reduced respiratory muscle capacity as a consequence of unfavorable pre-stretching of respiratory muscles fibers [33]. COPD as an underlying disease has already been described as a risk factor for unsuccessful weaning at LTACs [17, 34], although existing data are contradictory [8, 9]. As in patients with obesity, the presence of COPD in intubated patients is an independent risk factor for extubation failure [6], with similar recommendations for prophylactic use of NIV [35]. Both in COPD and obesity, the key predictor of increased weaning failure rate appears to be pre-existing increased WOB, accompanied by reduced respiratory muscle capacity potentially further exacerbated by factors such as controlled mechanical ventilation, sepsis, or steroids in the course of ICU treatment [36].

In the present study, the inability to spontaneously breathe for 30 min was independently associated with long-term ventilator dependency. Similar results were found in a study from Magnet and colleagues [18], in which hypercapnia at the end of a weaning trial was independently associated with weaning failure. Accordingly, in intubated patients, clinical signs of ventilatory insufficiency at the end of a weaning trial (i.e., increased rapid shallow breathing index or hypercapnia) are predictive of extubation failure [6] and, if they occur after a few minutes of spontaneous breathing, even predictive of the outcome of a weaning trial [37].

The finding that a lower P_a_CO_2_ on mechanical ventilation was an independent predictor of successful weaning is more difficult to explain. So far, this variable has been assessed in a single study, in which intubated patients with a successful weaning trial had significantly lower P_a_CO_2_ values on mechanical ventilation compared to patients who failed the SBT [38]. In the present study, although the amount of ventilatory assistance (IPAP, DP, and MP) was lower in successfully weaned patients, they had lower P_a_CO_2_ values on mechanical ventilation. In addition to the lower LTC, the reason for this may be the observed reduced VR, which is an expression of a more favorable ratio of CO_2_ production to MP, necessary to provide adequate gas exchange. In weaning failure patients, in order to achieve comparable mean P_a_CO_2_ values, it would be necessary to adjust ventilator settings further toward a higher MP (i.e., increasing IPAP or respiratory rate), and this in turn, considering the present results, appears to be associated with unsuccessful ventilator weaning.

Surprisingly, female sex was found to be independently associated with failure of the SBT and failure of prolonged weaning. There is no clear explanation for this finding; however, in one study, female gender has been identified as an independent risk factor for the occurrence of intensive care unit-acquired paresis, which in turn is associated with prolonged duration of mechanical ventilation [39]. In addition, differences in the mechanical properties of the respiratory system, most likely due to reduced lung volumes (total lung capacity and vital capacity) associated with both lower body height and female gender per se [40], ultimately leading to reduced LTC in the present study, may explain the observed differences in weaning failure rates between female and male patients.

Our study has several limitations. First, since this was a single-center study, the results are probably not transferable to other centers. Second, due to the difficulty in objectively measuring the amount of airway secretions and determining the type and frequency of different airway clearance techniques in a retrospective trial, we did not consider these important issues in terms of weaning outcome. Third, measurement of ventilator variables for calculating MP is ideally performed under deep sedation and muscle relaxation. Since patients admitted to the weaning center were neither sedated nor muscle relaxed, activation of respiratory or abdominal muscles cannot be ruled out without monitoring esophageal and gastric pressure [41]. This could either have led to an increase or decrease in VTi and minute ventilation, with a consequent distortion of LTC and MP. The same applies to activation of the diaphragm on assisted ventilation, although the proportion of assisted ventilated patients was low (16%) and not different when comparing successfully and unsuccessfully weaned patients. Furthermore, a simplified equation was used for calculation of MP in the pressure-controlled ventilation mode; however, this still seems to be sufficiently accurate for most clinical situations [24] and will probably facilitate application in routine practice.

## Conclusions

In the present study, the derived predictors of weaning point to a crucial role of the workload imposed on respiratory muscles during spontaneous breathing. Individuals on prolonged mechanical ventilation should not only be classified according to underlying diseases, but also by parameters that quantify the workload imposed on respiratory muscles during spontaneous breathing. Measurement of LTC-MP could be easily implemented in routine practice and may help clinicians in guiding the weaning process (e.g., to control the degree of daily extension of spontaneous breathing) or to identify patients at high risk for weaning failure at an early stage. Finally, LTC-MP may also be useful in intubated patients as a predictor of extubation failure or the outcome of a weaning trial and may help decision-making for prophylactic use of NIV. Further prospective studies are required to confirm the present results.

## Supplementary information

**Additional file 1.** Supplemental methods: Definitions of ventilator variables, respiratory indices, and weaning outcome measures; **Table S1.** [Comparison of female and male patients].

## Data Availability

The datasets used and/or analyzed during the current study are available from the corresponding author on reasonable request.

## Abbreviations

95% CI: 95% confidence interval
aBGA: Arterial blood gas analysis
APACHE II: Acute Physiology and Chronic Health Evaluation (score) 2
BF: Breathing frequency
BMI: Body mass index
bpm: Beats per minute
COPD: Chronic obstructive pulmonary disease
DP: Driving pressure
F_i_O_2_: Fraction of inspired oxygen
FRC: Functional residual capacity
HMV: Home mechanical ventilation
IMV: Invasive mechanical ventilation
IPAP: Inspiratory positive airway pressure
LTAC: Long-term acute care facility
LTC: Dynamic lung-thorax compliance
LTC-MP: Mechanical power normalized to lung thorax compliance
LTOT: Long-term oxygen therapy
MP: Mechanical power
NIV: Non-invasive ventilation
OR: Odds ratio
PBW: Predicted body weight
PBW-MP: Mechanical power normalized to predicted body weight
PEEP: Positive end-expiratory pressure
P-V curve: Pressure-volume curve of the respiratory system
SBT: Spontaneous breathing trial
SD: Standard deviation
VE: Minute ventilation
VE-MP: Mechanical power normalized to minute ventilation
VILI: Ventilator-induced lung injury
VR: Ventilatory ratio
VTi: Inspiratory tidal volume
WOB: Work of breathing

## References

[1] Torres A, Gatell JM, Aznar E (1995). Re-intubation increases the risk of nosocomial pneumonia in patients needing mechanical ventilation. Am J Respir Crit Care Med.

[2] Girou E, Schortgen F, Delclaux C (2000). Association of noninvasive ventilation with nosocomial infections and survival in critically ill patients. JAMA.

[3] Esteban A, Anzueto A, Frutos F (2002). Characteristics and outcome in adult patients receiving mechanical ventilation. JAMA.

[4] Ely W, Baker AM, Dunagan DP (1996). Effect on the duration of mechanical ventilation of identifying patients capable of breathing spontaneously. N Engl J Med.

[5] Boles JM, Bion J, Connors A (2007). Weaning from mechanical ventilation. Eur Respir J.

[6] Baptistella AF, Sarmento FJ, Ribeiro da Silva K (2018). Predictive factors of weaning from mechanical ventilation and extubation outcome: a systematic review. J Crit Care.

[7] Schönhofer B, Euteneuer S, Nava S (2002). Survival of mechanically ventilated patients admitted to a specialised weaning centre. Intensive Care Med.

[8] Pilcher DV, Bailey MJ, Treacher DF (2005). Outcomes, cost and long term survival of patients referred to a regional weaning centre. Thorax.

[9] Bonnici DM, Sanctuary T, Warren A (2016). Prospective observational cohort study of patients with weaning failure admitted to a specialist weaning, rehabilitation and home mechanical ventilation centre. BMJ Open.

[10] Jubran A, Grant BJB, Duffner LA (2019). Long-term outcome after prolonged mechanical ventilation. Am J Respir Crit Care Med.

[11] Purro A, Appendini L, De Gaetano A (2000). Physiologic determinants of ventilator dependence in long-term mechanically ventilated patients. Am J Respir Crit Care Med.

[12] Carlucci A, Ceriana P, Prinianakis G (2009). Determinants of weaning success in patients with prolonged mechanical ventilation. Crit Care.

[13] Nava S, Rubini F, Zanotti E (1994). Survival and prediction of successful ventilator weaning in COPD patients requiring mechanical ventilation for more than 21 days. Eur Respir J.

[14] Scheinhorn DJ, Hassenpflug M, Artinian BM (1995). Predictors of weaning after 6 weeks of mechanical ventilation. Chest.

[15] Gluck EH, Corigan L (1996). Predicting eventual success or failure to wean in patients receiving long-term mechanical ventilation. Chest.

[16] Hendra KP, Bonis PAL, Joyce-Brady M (2003). Development and prospective validation of a model for predicting weaning in chronic ventilator dependent patients. BMC Pulm Med.

[17] Mamary AJ, Kondapaneni S, Vance GP (2011). Survival in patients receiving prolonged ventilation: factors that influence outcome. Clin Med Insights Circ Respir Pulm Med.

[18] Magnet FS, Bleichroth H, Huttmann SE (2018). Clinical evidence for respiratory insufficiency type II predicts weaning failure in long-term ventilated, tracheotomised patients: a retrospective analysis. J Intensive Care.

[19] Schönhofer B, Geiseler J, Dellweg D (2014). Prolonged weaning: S2k-guideline published by the German Respiratory Society. Pneumologie.

[20] Jubran A, Grant BJ, Duffner LA (2013). Effect of pressure support vs unassisted breathing through a tracheostomy collar on weaning duration in patients requiring prolonged mechanical ventilation: a randomized trial. JAMA.

[21] Okabe Y, Asaga T, Bekku S (2018). Lung-thorax compliance measured during a spontaneous breathing trial is a good index of extubation failure in the surgical intensive care unit: a retrospective cohort study. J Intensive Care.

[22] Gattinoni L, Tonetti T, Cressoni M (2016). Ventilator-related causes of lung injury: the mechanical power. Intensive Care Med.

[23] Serpa Neto A, Deliberato RO, Johnson AEW (2018). Mechanical power of ventilation is associated with mortality in critically ill patients: an analysis of patients in two observational cohorts. Intensive Care Med.

[24] Becher T, van der Staay M, Schädler D (2019). Calculation of mechanical power for pressure-controlled ventilation. Intensive Care Med.

[25] Sinha P, Fauvel NJ, Singh S (2009). Ventilatory ratio: a simple bedside measure of ventilation. Br J Anaesth.

[26] Sinha P, Fauvel NJ, Pradeep S (2013). Analysis of ventilatory ratio as a novel method to monitor ventilatory adequacy at the bedside. Crit Care.

[27] Zhang Z, Zheng B, Niu N (2019). Mechanical power normalized to predicted body weight as a predictor of mortality in patients with acute respiratory distress syndrome. Intensive Care Med.

[28] Fiastro JF, Habib MP, Shon BY (1988). Comparison of standard weaning parameters and the mechanical work of breathing in mechanically ventilated patients. Chest.

[29] Jubran A, Grant BJB, Laghi F (2005). Weaning prediction: esophageal pressure monitoring complements readiness testing. Am J Respir Crit Care Med.

[30] Banner MJ, Euliano NR, Martin AD (2012). Noninvasive work of breathing improves prediction of post-extubation outcome. Intensive Care Med.

[31] De Jong A, Chanques G, Jaber S (2017). Mechanical ventilation in obese ICU patients: from intubation to extubation. Crit Care.

[32] El Solh AA, Aquilina A, Pineda L (2006). Noninvasive ventilation for prevention of post-extubation respiratory failure in obese patients. Eur Respir J.

[33] Gea J, Agusti A, Roca J (2013). Pathophysiology of muscle dysfunction in COPD. J Appl Physiol.

[34] Dasgupta A, Rice R, Mascha E (1999). Four-year experience with a unit for long-term ventilation (respiratory special care unit) at the Cleveland Clinic Foundation. Chest.

[35] Davidson AC, Banham S, Elliott M (2016). BTS/ICS guideline for the ventilatory management of acute hypercapnic respiratory failure in adults. Thorax.

[36] Demoule A, Jung B, Prodanovic H (2013). Diaphragm dysfunction on admission to the intensive care unit. Prevalence, risk factors, and prognostic impact–a prospective study. Am J Respir Crit Care Med.

[37] Yang KL, Tobin MJ (1991). A prospective study of indexes predicting the outcome of trials of weaning from mechanical ventilation. N Engl J Med.

[38] Farghaly S, Galal M, Hasan AA (2015). Brain natriuretic peptide as a predictor of weaning from mechanical ventilation in patients with respiratory illness. Aust Crit Care.

[39] De Jonghe B, Sharshar T, Lefaucheur JP (2002). Paresis acquired in the intensive care unit. A prospective multicenter study. JAMA.

[40] Quanjer PH, Stanojevic S, Cole TJ (2012). Multi-ethnic reference values for spirometry for the 3–95-yr age range: the global lung function 2012 equations. Eur Respir J.

[41] Appendini L, Purro A, Patessio A (1996). Partitioning of inspiratory muscle workload and pressure assistance in ventilator-dependent COPD patients. Am J Respir Crit Care Med.

