# Supplementary material for: Variables predicting weaning outcome in prolonged mechanically ventilated tracheotomized patients: a retrospective study
Source: J Intensive Care. 2020 Feb 21;8:19. doi: 10.1186/s40560-020-00437-4 (PMC7035768; doi:10.1186/s40560-020-00437-4)
Supplement: Supplementary file 1 — Additional file 1. Supplemental methods: Definitions of ventilator variables, respiratory indices, and weaning outcome measures; Table S1. [Comparison of female and male patients]. [file 40560_2020_437_MOESM1_ESM.pdf]

Variables predicting weaning outcome in prolonged mechanically ventilated tracheotomized patients: a retrospective study

Alessandro Ghiani, MD; Joanna Paderewska, MD; Alexandros Sainis, MD; Alexander Crispin, MD; Swenja Walcher, RT; and Claus Neurohr, MD.

Additional file 1

<u>Supplemental methods</u>

1. **Definitions of ventilator variables and respiratory indices (Page 2)**
2. **Definitions of weaning outcome measures (Page 3)**
3. **Table S1 (Page 4)**
4. **References (Page 6)**

Supplemental methods

1. Definitions of ventilator variables and respiratory indices

**PEEP**	Positive end-expiratory pressure (PEEP).
**IPAP**	Inspiratory positive airway pressure (IPAP) including PEEP.
**Driving pressure (DP)**	Driving pressure (DP) was calculated using IPAP and PEEP:<br><br>**DP (cmH_2O) = IPAP – PEEP**
**Dynamic lung-thorax compliance (LTC)**	Dynamic lung-thorax compliance (LTC) was calculated using tidal volumes (VTi) and driving pressures:<br><br>**LTC (mL/cmH_2O) = VTi / DP**
**Mechanical power (MP)**	Mechanical power (MP) provided by the ventilator in the pressure-controlled mode was calculated using VTi, breathing frequency (BF), and IPAP [1]:<br><br>**MP (J/min) = 0.098 x VTi x BF x IPAP**<br><br>With each breath delivered by the ventilator a certain amount of energy (J) is transferred to the patients` respiratory system. This energy is mainly used to overcome resistance of the airways and to inflate the lungs and expand the thoracic cage. MP (J/min) unifies variables known to be related to development of ventilator-induced lung injury [2], and is independently associated with worse outcomes in patients receiving invasive ventilation [3].
**Mechanical power normalized to predicted body weight (PBW-MP)**	MP normalized to predicted body weight (PBW-MP) provided by the ventilator was calculated using MP and predicted body weight (PBW):<br><br>**PBW-MP (J/min/kg) = MP / PBW = MP / (body length [cm]–100 [cm])**<br><br>PBW-MP is independently associated with increased mortality in ARDS [4].
**Mechanical power normalized to lung-thorax compliance (LTC-MP)**	Mechanical power normalized to lung-thorax compliance (LTC-MP) was calculated using MP and dynamic lung-thorax compliance (LTC) [4]:<br><br>**LTC-MP (J/min * cmH_2O/mL) = MP / LTC**
**Mechanical power normalized to minute ventilation (VE-MP)**	Mechanical power normalized to minute ventilation (= mechanical work) provided by the ventilator was calculated using mechanical power (MP) and minute ventilation (VE):<br><br>**VE-MP (J/L) = MP / VE**
**Ventilatory ratio (VR)**	Ventilatory ratio (VR) is a surrogate of pulmonary dead space fraction and a simple bedside index of impaired efficiency of ventilation [5-6]:<br><br>**$VR = VE_{measured} \times P_aCO_{2measured} / VE_{predicted} \times P_aCO_{2ideal}$**<br><br>$VE_{measured}$ is the measured minute ventilation (mL/min), $P_aCO_{2measured}$ is the measured arterial pressure of carbon dioxide (mmHg), $VE_{predicted}$ is the predicted minute ventilation calculated as predicted body weight x 1000 (mL/min), and P_aCO2_{ideal} is the expected arterial pressure of carbon dioxide in normal lungs if ventilated with the predicted minute ventilation. P_aCO_{2ideal} is set as 37.5 mmHg (5 kPa) for all patients. VR is a unitless ratio, and a value approximating 1 would represent normal ventilating lungs.
**Oxygen flow on MV**	Liters of oxygen per minute fed to the breathing tube.

2. Definitions of weaning outcome measures

**Weaning success**	Weaning success was defined as permanent spontaneous breathing for more than 7 days without concomitant clinical or laboratory signs of chronic ventilatory insufficiency.
**Weaning failure**	Weaning failure was defined as either transition to non-invasive home ventilation (NIV), invasive home ventilation (HMV-IMV), or death on ventilation in the course of weaning.
**Weaning duration**	Time from 1st SBT upon admission to the weaning unit to the point at which weaning was completed.<br><br>**Weaning success:** Equally to the time of the last mechanical ventilation episode with the following permanent spontaneous breathing.<br><br>**Weaning failure:** Equally to either the time to transition to NIV or HMV-IMV (this was at the discretion of the treating physician), or death on ventilation in the course of weaning.<br><br>➢ In the case of NIV, time to transition to NIV is not always the same as the time to decannulation. If decannulation has been delayed for other medical reasons, such as repeated bronchoscopic interventions for resection of subglottic tracheal stenosis prior to decannulation, then the time was chosen as the end of the weaning process, from which, due to the ventilatory capacity, a switch to NIV was considered.
**Weaning unit length of stay**	Time from admission to the weaning center until discharge from the weaning unit.
**Hospital length of stay**	Time from admission to the weaning center until discharge from the hospital.
**Spontaneous breathing on discharge**	Number of hours of spontaneous breathing on discharge from the hospital, equals to 24 hours minus the number of hours of mechanical ventilation per day.<br><br>Duration of spontaneous breathing of deceased patients was rated at 0 hours.
**LTOT on discharge**	Long-term oxygen therapy (LTOT) on discharge from the hospital.
**Hospital mortality**	Proportion of patients deceased during their hospital stay.

3. Table S1: Comparison of female and male patients

Clinical characteristics	All patients (n = 263)	Female gender (n = 102)	Male gender (n = 161)	P value[a]
Age (years)	70.9 (± 11.0)	70.5 (± 12.2)	71.1 (± 10.1)	0.317[b]
Weight (kg)	78.4 (± 17.6)	76.1 (± 21.1)	80.0 (± 14.9)	**0.041[b]**
Height (m)	1.72 (± 0.08)	1.66 (± 0.08)	1.76 (± 0.06)	**< 0.001[b]**
Body mass index (kg/m^2)	26.4 (± 6.2)	27.5 (± 7.8)	25.8 (± 4.9)	**0.016[b]**
Obesity (BMI ≥ 30 kg/m^2)	54 (20.5)	27 (26.5)	27 (16.8)	0.058[c]
Smoking history	120 (45.6)	38 (37.3)	82 (50.9)	**0.030[c]**
APACHE-II (points)	17.7 (± 5.1)	17.6 (± 4.5)	17.8 (± 5.4)	0.355[b]
Albumin (g/dL)	2.0 (± 0.5)	2.1 (± 0.4)	1.9 (± 0.5)	0.213[b]
Ventilator days on admission (days)	26.7 (± 21.6)	24.7 (± 19.3)	28.0 (± 22.9)	0.118[b]
Time from Intubation to tracheostomy (days)	10.6 (± 6.7)	10.5 (± 7.3)	10.6 (± 6.3)	0.431[b]
ECLA	11 (4.2)	2 (2.0)	9 (5.6)	0.151[c]
**Cause of acute respiratory failure**				
Pneumonia	77 (29.3)	31 (30.4)	46 (28.6)	0.752[c]
Surgery	74 (28.1)	24 (23.5)	50 (31.1)	0.186[c]
Sepsis (including septic shock)	29 (11.0)	7 (6.9)	22 (13.7)	0.086[c]
Acute exacerbation of COPD	26 (9.9)	14 (13.7)	12 (7.5)	0.097[c]
Cardiopulmonary resuscitation	22 (8.4)	11 (10.8)	11 (6.8)	0.259[c]
Cardiac failure	8 (3.0)	4 (3.9)	4 (2.5)	0.508[c]
Trauma	5 (1.9)	0 (0.0)	5 (3.1)	0.160[d]
Other	22 (8.4)	11 (10.8)	11 (6.8)	0.259[c]
**Comorbidities**				
Charlson comorbidity index (points)	6.6 (± 2.4)	6.1 (± 2.5)	6.9 (± 2.4)	**0.005[b]**
Coronary artery disease	89 (33.8)	23 (22.6)	66 (41.0)	**0.002[c]**
COPD	65 (24.7)	27 (26.5)	38 (23.6)	0.599[c]
Diabetes mellitus	82 (31.2)	27 (26.5)	55 (34.2)	0.190[c]
Chronic heart failure	53 (20.2)	11 (10.8)	42 (26.1)	**0.002[c]**
Renal insufficiency (GFR < 60 mL/min)	74 (28.1)	30 (29.4)	44 (27.3)	0.714[c]
Hemodialysis on admission	34 (12.9)	17 (16.7)	17 (10.1)	0.150[c]
Malignancy	37 (14.1)	12 (11.8)	25 (15.5)	0.392[c]
Hepatopathy	13 (4.9)	3 (2.9)	10 (6.2)	0.233[c]
Interstitial lung disease	12 (4.6)	4 (3.9)	8 (5.0)	0.692[c]
Neuromuscular disease	12 (4.6)	4 (3.9)	8 (5.0)	0.692[c]
**Ventilator variables**				
IPAP (cmH$_2$O)	23.4 (± 4.8)	24.1 (± 4.6)	22.9 (± 4.8)	**0.020[b]**
PEEP (cmH$_2$O)	5.9 (± 1.2)	6.0 (± 1.6)	5.8 (± 0.9)	0.133[b]
DP (cmH$_2$O)	17.5 (± 4.5)	18.1 (± 4.3)	17.1 (± 4.6)	**0.030[b]**
VTi (mL)	534 (± 82)	514 (± 80)	547 (± 80)	**< 0.001[b]**
BF (breaths/min)	16.2 (± 2.5)	16.1 (± 2.3)	16.3 (± 2.6)	0.238[b]
VE (L/min)	8.6 (± 1.8)	8.3 (± 1.8)	8.9 (± 1.8)	**0.004[b]**
Oxygen flow on MV (L/min)	2.6 (± 1.8)	2.4 (± 1.8)	2.7 (± 1.7)	0.101[b]
**Respiratory indices**				
LTC (mL/cmH$_2$O)	32.8 (± 10.8)	29.6 (± 7.8)	34.8 (± 11.9)	**< 0.001[b]**
Mechanical power (J/min)	19.8 (± 5.9)	19.7 (± 6.0)	19.9 (± 5.8)	0.375[b]
*PBW-MP (J/min/kg PBW * 10^{-3})*	277 (± 87)	299 (± 98)	262 (± 76)	**< 0.001[b]**
*LTC-MP (J/min * cmH$_2$O/mL * 10^{-3})*	685 (± 348)	723 (± 349)	661 (± 345)	0.077[b]
VE-MP (J/L)	2.29 (± 0.47)	2.36 (± 0.45)	2.25 (± 0.47)	**0.021[b]**

Ventilatory ratio	1.14 (± 0.27)	1.16 (± 0.30)	1.12 (± 0.26)	0.099[b]
**First SBT**				
Time from admission to first SBT (days)	2.5 (± 4.2)	2.7 (± 4.0)	2.4 (± 4.4)	0.308[b]
Hemoglobin on first SBT (g/dL)	8.8 (± 1.2)	8.7 (± 1.2)	8.8 (± 1.2)	0.153[b]
P_aCO_2 on aBGA				
P_aCO_2 on MV pre-SBT (mmHg)	35.7 (± 5.0)	35.0 (± 4.9)	36.1 (± 5.0)	**0.038[b]**
P_aCO_2 post-SBT (mmHg)	39.1 (± 6.5)	39.4 (± 7.4)	38.9 (± 5.8)	0.285[b]
ΔP_aCO_2 (pre-/post-SBT)	3.4 (5.5)	4.6 (± 6.2)	2.8 (± 4.9)	**0.006[c]**
pH on aBGA				
pH on MV pre-SBT	7.48 (± 0.05)	7.49 (± 0.05)	7.48 (± 0.05)	**0.015[b]**
pH post-SBT	7.45 (± 0.05)	7.45 (± 0.06)	7.45 (± 0.05)	0.438[b]
ΔpH (pre-/post-SBT)	–0.03 (± 0.05)	–0.05 (± 0.06)	–0.03 (± 0.05)	**0.007[b]**
P_aO_2 on aBGA				
P_aO_2 on MV pre-SBT (mmHg)	81.4 (± 18.7)	81.2 (± 18.6)	81.5 (± 18.8)	0.456[b]
P_aO_2 post-SBT (mmHg)	74.3 (± 17.2)	78.2 (± 20.8)	72.1 (± 14.4)	**0.005[b]**
ΔP_aO_2 (pre-/post-SBT)	6.9 (22.3)	2.0 (± 24.7)	9.7 (± 20.2)	**0.005[b]**
Duration of first SBT (min)	27.0 (± 7.5)	25.3 (± 9.1)	28.0 (± 6.1)	**0.003[b]**
Reason for failure of first SBT	72 (27.4)	36 (35.3)	36 (22.4)	**0.023[c]**
Acidosis post-SBT	10 (3.8)	4 (3.9)	6 (3.7)	0.936[d]
Hypercapnia post-SBT	37 (14.1)	16 (15.7)	21 (13.0)	0.514[c]
Premature termination of SBT	42 (16.0)	23 (22.5)	19 (11.8)	**0.020[c]**
**Weaning outcome measures**				
Weaning success	126 (47.9)	39 (38.2)	87 (54.0)	**0.012[c]**
Weaning failure	137 (52.1)	63 (61.8)	74 (46.0)	**0.012[c]**
HMV-NIV	50 (19.0)	23 (22.5)	27 (16.8)	0.245[c]
HMV-IMV	74 (28.1)	35 (34.3)	39 (24.2)	0.076[c]
Death on MV	13 (4.9)	5 (4.9)	8 (5.0)	0.981[c]
Weaning duration from first SBT (days)	22.9 (± 16.1)	26.1 (± 19.2)	20.9 (± 13.4)	**0.006[b]**
Duration of mechanical ventilation (days)	52.2 (± 28.6)	53.5 (± 29.6)	51.3 (± 27.9)	0.274[b]
SB on discharge from hospital (hours per day)[d]	16.1 (± 8.9)	15.4 (± 8.4)	16.6 (± 9.2)	0.129[b]
LTOT on discharge	183 (78.9)	78 (84.8)	105 (75.0)	0.053[c]
Weaning unit-LOS (days)	48.3 (± 28.5)	51.2 (± 31.1)	46.4 (± 26.5)	0.089[b]
Hospital-LOS (days)	52.8 (± 28.6)	55.5 (± 31.4)	51.1 (± 26.6)	0.116[b]
Hospital mortality	38 (14.5)	12 (11.8)	26 (16.1)	0.324[c]

Legend

Continuous variables are presented as mean values (± standard deviation); categorical variables are presented as number (%).

a: P value for differences between female and male patients

b: Student`s *t*-test

c: Chi square test

d: Fisher`s Exact test

Abbreviations: BMI, body mass index; APACHE-II, Acute Physiology and Chronic Health Evaluation II score; ECLA, extracorporeal lung assistance (in acute respiratory failure); COPD, chronic obstructive pulmonary disease; GFR, glomerular filtration rate; IPAP, inspiratory positive pressure ventilation; PEEP, positive end-expiratory pressure; DP, driving pressure; VTi, inspiratory tidal volume; BF, breathing frequency; VE, minute ventilation; MV, mechanical ventilation; LTC, dynamic lung-thorax compliance; MP, mechanical power; J, joule; PBW-MP, mechanical power normalized to predicted body weight; PBW, predicted body weight; LTC-MP, mechanical power normalized to dynamic lung-thorax compliance; VE-MP, mechanical power normalized to minute ventilation; aBGA, arterial blood gas analysis; SBT, spontaneous breathing trial; HMV-NIV, home mechanical ventilation-non-invasive mechanical ventilation; HMV-IMV, home mechanical ventilation-invasive mechanical ventilation; IMV, invasive mechanical ventilation; SB, spontaneous breathing; LTOT, long-term oxygen therapy; SBT, spontaneous breathing trial; LOS, length of stay.

4. References

1. Becher T, van der Staay M, Schädler D, et al. Calculation of mechanical power for pressure-controlled ventilation. Intensive Care Med 2019;45:1321–23
2. Gattinoni L, Tonetti T, Cressoni P, et al. Ventilator-related causes of lung injury: the mechanical power. Intensive Care Med 2016;42:1567–75
3. Serpa Neto A, Deliberato RO, Johnson AEW, et al. Mechanical power of ventilation is associated with mortality in critically ill patients: an analysis of patients in two observational cohorts. Intensive Care Med 2018;44:1914–22
4. Zhang Z, Zheng B, Niu N, et al. Mechanical power normalized to predicted body weight as a predictor of mortality in patients with acute respiratory distress syndrome. Intensive Care Med 2019;45:856–64
5. Sinha P, Fauvel NJ, Singh S, et al. Ventilatory ratio: a simple bedside measure of ventilation. Br J Anaesth 2009;102:692–97
6. Sinha P, Fauvel NJ, Pradeep S, et al. Analysis of ventilatory ratio as a novel method to monitor ventilatory adequacy at the bedside. Crit Care 2013;17:R34
